# Pharmacokinetic Delivery and Metabolizing Rate of Nicardipine Incorporated in Hydrophilic and Hydrophobic Cyclodextrins Using Two-Compartment Mathematical Model

**DOI:** 10.1155/2013/131358

**Published:** 2013-12-03

**Authors:** Sergey Shityakov, Carola Förster

**Affiliations:** Department of Anaesthesia and Critical Care, University of Würzburg, 97080 Würzburg, Germany

## Abstract

The dispersion routes of cyclodextrin complexes with nicardipine (NC), such as hydrophilic hydroxypropyl-*β*-cyclodextrin (NC/HP*β*CD) and hydrophobic triacetyl-*β*-cyclodextrin (NC/TA*β*CD), through the body for controlled drug delivery and sustained release have been examined. The two-compartment pharmacokinetic model described the mechanisms of how the human body handles with ingestion of NC-cyclodextrin complexes in gastrointestinal tract (GI), distribution in plasma, and their metabolism in the liver. The model showed that drug bioavailability was significantly improved after oral administration of cyclodextrin complexes. The mathematical significance of this study to predict nicardipine delivery using pharmacokinetic two-compartment mathematical model with linear ordinary differential equations (ODE) approach represents a valuable tool to emphasize its effectiveness and metabolizing rate and diminish the side effects.

## 1. Introduction

Formulation of drugs with different drug carrier materials to control and improve drug release is a rapidly growing area in the pharmaceutical sciences. One example of this application is the usage of cyclodextrins (CDs) as excipients for a sustained release of various chemical compounds after their oral administration [1, 2]. Various mathematical models were proposed to study the CD release profiles and match them to the experimentally-determined kinetic data [3, 4]. Among those methods were Weibull distribution along with zero-order, Higuchi, and Korsmeyer-Peppas that best describe drug release phenomena with major applicability to the versatile experimental data [5, 6].

In this context, the experimental and theoretical approaches are increasing importance of the investigation of complexation mechanisms and pharmacokinetic properties of CD-drug conjugates, as well as their delivery mechanisms in the different compartments of the human body [7–9].

Nicardipine (NC) is a calcium-channel blocker used in the treatment of different cardiovascular and cerebral diseases; these diseases are mainly hypertension, subarachnoid hemorrhage, and acute strokes [10, 11]. This drug was used as a model to simulate the limited bioavailability effects (up to 40%) and rapid biotransformation in the liver with a very short half-life time [12].

The study of Fernandes and coauthors had reported that nicardipine release rate might be controlled by the formulation of this drug with hydroxypropyl-*β*-cyclodextrin (NC/HP*β*CD) and triacetyl-*β*-cyclodextrin (NC/TA*β*CD) derivatives [1]. The nicardipine delivery can be modeled by using two-compartment pharmacokinetic model developed previously in the works of Spitznagel and Shonkwiler and Herod [13, 14]. This approach was already implemented successfully to investigate the transfer and elimination of lead compound from blood and bone tissues and assess the influence of bone-lead depots [15].

The current study attempts to predict the nicardipine release rate based on its formulation with hydrophilic and hydrophobic cyclodextrins in the gastrointestinal tract (GI) and plasma using pharmacokinetic two-compartment mathematical model with linear ordinary differential equations (ODE) approach. Additionally, the metabolizing rate analysis was conducted, and the results were compared to the experimentally-acquired data using the referred model.

## 2. Computational Methods

The experimental pharmacokinetic parameters, such as maximum plasma concentration (*C*_max⁡_), the time to reach *C*_max⁡_ (*T*_max⁡_), and the elimination half-life (*t*_1/2_  ) together with dissolution profiles, were taken from *in vivo* study of Fernandes and coauthors [1]. The authors analyzed the drug release rate in rabbits (*n* = 6) using high performance liquid chromatography with UV detection.

To simulate the nicardipine dose-dependent release and its metabolic degradation, we used computer linear algebra algorithms implemented in the Maple 15 software package (Maplesoft GmbH, Aachen, Germany). The pharmacokinetic two-compartment model included two different compartments: the first compartment ({*x*(*t*)}  or  *dx*/*dt*) describes the drug concentration in the GI, and the second compartment ({*y*(*t*)}  or  *dy*/*dt*) describes its concentration in plasma in dosage-dependent manner (long-range) by following differential equation:

(1) $$\begin{array}{c} \frac{dx}{dt}=\frac{D\left(t\right)}{\text{DR}}-xt_{1/2}, \end{array}$$

(2) $$\begin{array}{c} \frac{dy}{dt}=xt_{1/2}-yt_{t_{1/2}}, \end{array}$$

where *D*(*t*) is the drug dosage in *μ*g/mL, DR is the dissolution rate, and *t*_1/2_ value is the half-life parameter either for the GI or plasma. The dissolution rate was calculated from experimentally-determined profiles in the acidic medium (pH 1.2) for nicardipine and its complexes as

(3) $$\begin{array}{c} \text{DR}=\frac{t_{\text{complex}}}{t_{\text{drug}}}, \end{array}$$

where *t*_complex_ and *t*_drug_ are the dissolution time in minutes either for nicardipine or its complexes in the acidic environment.

The Runge-Kutta method was used to solve numerically the ODE by using a trial step at the midpoint of an interval to cancel out lower-order error terms [16, 17]. This method was already proven for a variety of pharmacokinetic simulations as a very robust technique [18–20].

The ODE approach was also applied to solve the first-order irreversible series of drug translocations and modifications to obtain symbolically the exponential matrix as a function of time [21]; the independent variable uses the following scheme:

(4) Drug  or  Complex(GI)→k
GI
Drug  or  Complex(Plasma)→kPlasmaMetabolites  (Plasma),

where *k*_ GI_ and *k*_Plasma_ are the estimated rate constants either in the GI or plasma.

Therefore, in the Maple software package for a linear system of *n* simultaneous coupled first-order differential equations, *Y* is *n* × 1 matrix, *A* is a *n* × *n* matrix, and exp⁡(*At*) is a *n* × *n* matrix, and so on [21]. The governing equation for this reaction can be represented as

(5) $$\begin{array}{c} \frac{dC_{A}}{dt}=-{k_{\text{GI}}C}_{A}, \end{array}$$

(6) $$\begin{array}{c} \frac{dC_{B}}{dt}=k_{\text{GI}}C_{A}-k_{\text{Plasma}}C_{B}, \end{array}$$

where *C*_*A*_ and *C*_*B*_ are the concentrations for analyzing species either in the GI or plasma (short-range). The *C*_*A*_ value was set to 1.0 ng/mL as the initial concentration. The concentration of metabolite species *C*_*C*_ at any given time (*C*_*C*_(*t*)) is

(7) $$\begin{array}{c} C_{C}\left(t\right)={1-C}_{A}\left(t\right)-C_{B}\left(t\right). \end{array}$$

The estimated rate constants (Table 1) were calculated from experimentally-determined *t*_1/2_ values for nicardipine and its complexes as

(8) $$\begin{array}{c} k=\frac{\ln2}{t_{1/2}}. \end{array}$$

For a compartment model, we used the diagonal terms of the matrix and the column sums, which were negative or zero. Therefore, the solution has a tendency to be driven by drug dosage (*D*(*t*)).

## 3. Results and Discussion

According to our model, nicardipine and its CD complexes were taken orally at periodic time intervals to provide a pulse of dosage delivered to the gastrointestinal tract. From there, the substances entered into the bloodstream in concentration-dependent and dissolution-dependent manner. The drug was assumed to be distributed into the bloodstream compartment after its partial or complete elimination from the GI implying the bioexponential drug disposition and distribution.

To establish the absorption rate as the *t*_1/2_ value of NC, NC/HP*β*CD, and NC/TA*β*CD substances, we specified dissolution-dependent drug dose taking into account that the NC dissolution was observed within 180 min due to high partitioning coefficient (log⁡⁡*P* = 3.94) [22], after 15 min for NC/HP*β*CD and only after 480 min for NC/TA*β*CD, respectively. The retention time of the last compound was explained by the hydrophobic character of this complex. Meanwhile, the hydrophilic character of HP*β*CD with very low log⁡⁡*P*(−14.57) Hazai et al. [23] ensured a significant enhancement of the low NC dissolution and solubility because of surfactant-like properties of the carrier diminishing drug-medium interfacial tension [24, 25]. The starting *D*(*t*) value was calculated for each substance as *D*(*t*)/12 for NC and *D*(*t*)/32 for NC/TA*β*CD, following the logic that these substances have 12- and 32-fold decrease in dissolution in comparison to hydrophilic cyclodextrin formulation. However, the final dosage value for NC/HP*β*CD was actually devised from precalculated concentration (0.49 *μ*g/mL) using initial *D*(*t*). Further, this value was diminished to *D*(*t*)/12 to fit its predicted *C*_max⁡_ parameter to the experimentally-determined results (39.65 ± 1.11 *μ*g/mL).

**Figure 1:**
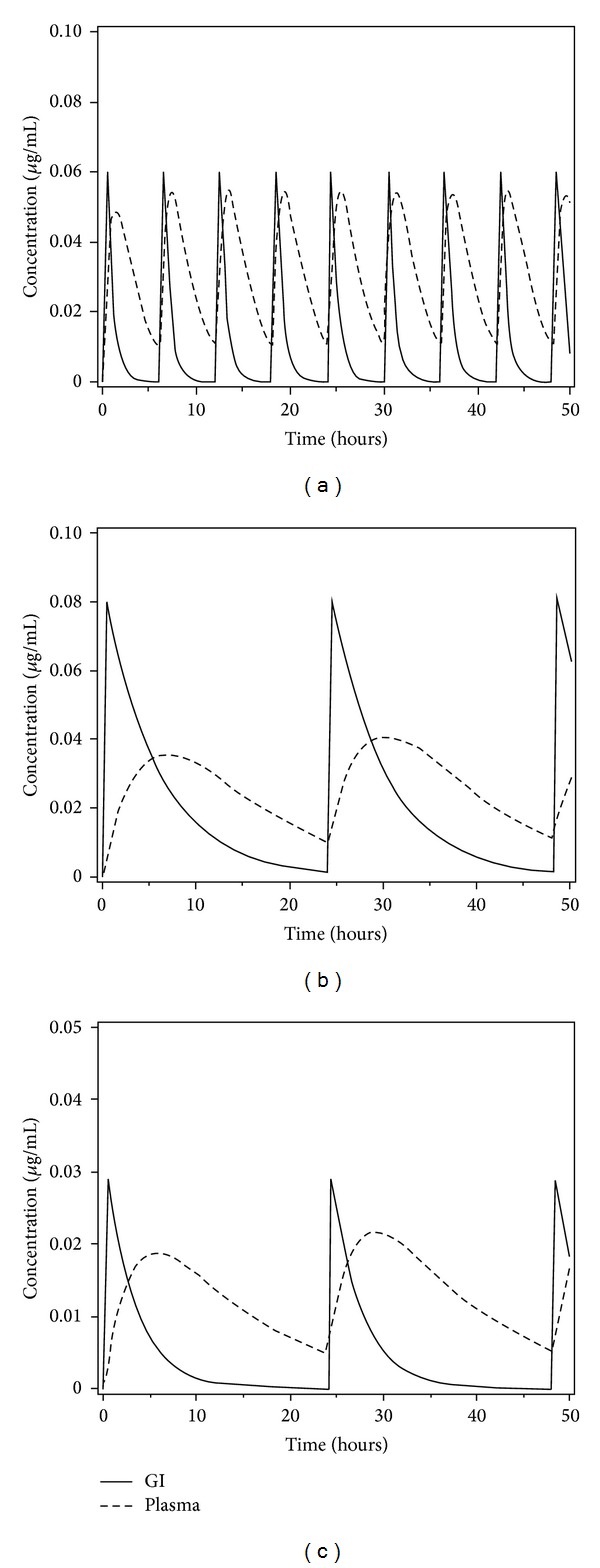
Loading of long-range GI and plasma concentration-time profiles from a dosage regime for NC (a), NC/HP*β*CD (b), and NC/TA*β*CD (c) compounds after oral administration.

Since the maximal plasma levels of NC were already observed after 30 min of oral administration, we used a 30 min half-life in the GI tract for nicardipine such as a *t*_1/2_ of 2ln⁡⁡(2). For the NC/HP*β*CD and NC/TA*β*CD complexes, we used empirically determined *t*_1/2_ values of ln⁡⁡(2)/4 and ln⁡⁡(2)/2.2 since these compounds have much longer half-lives in the GI tract in comparison to NC [24]. The plasma *t*_1/2_ parameters were ln⁡⁡(2)/1.6 for NC, ln⁡⁡(2)/5.49 for NC/HP*β*CD, and ln⁡⁡(2)/7.38 for NC/TA*β*CD according to their experimentally-determined half-life rates [1]. We set *x*(0) = *y*(0) = 0 for initial conditions to adjust the starting point where no substance is present either in the GI tract or circulatory system. The Runge-Kutta method was implemented to produce Figures 1(a)–1(c) for the numerical solution of ODE using the corresponding Maple code (Algorithm 1).

**Figure 2:**
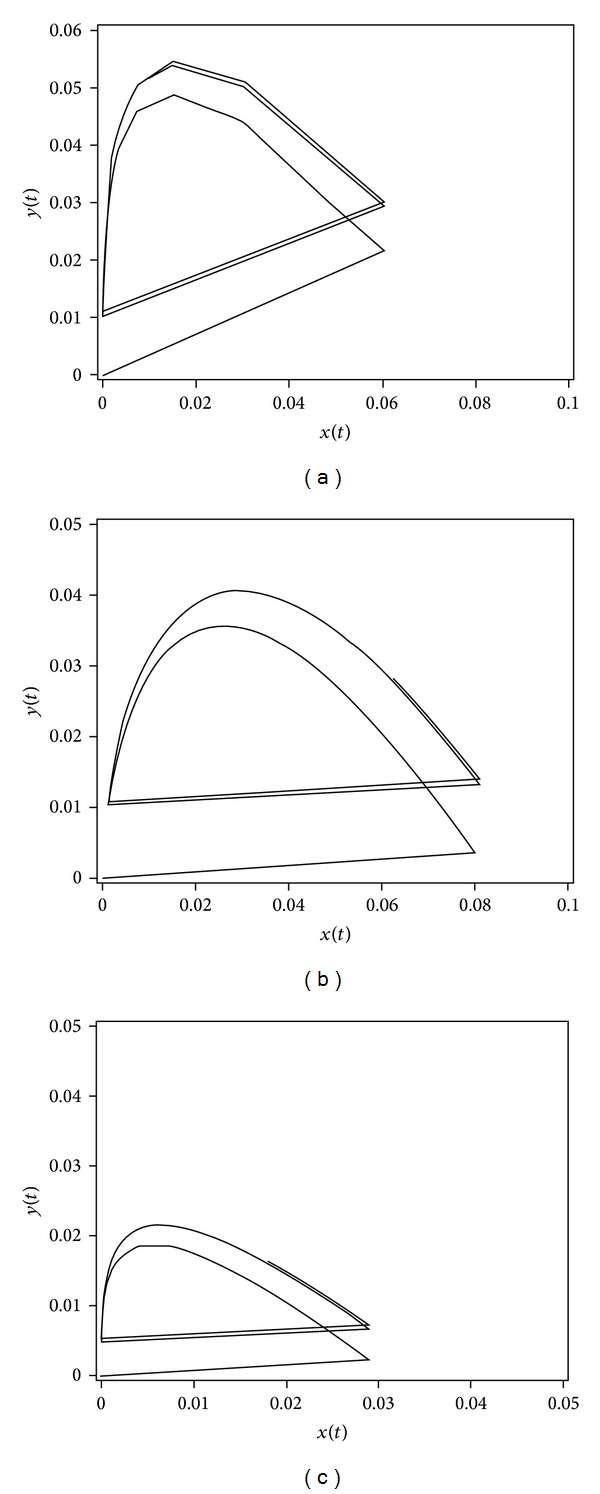
{*x*(*t*), *y*(*t*)} with limit cycle for NC, NC/HP*β*CD, and NC/TA*β*CD substances.

Figures 2(a)–2(c) show the limit cycles for the NC, NC/HP*β*CD, and NC/TA*β*CD substances that asymptotically tends to be periodic but not sinusoidal. The behavior of NC concentration-time profiles in the GI {*x*(*t*)} or plasma {*y*(*t*)} predicts an oscillating increase and decrease of the NC concentration. Interestingly, the NC concentration in the bloodstream was predicted to be oscillating every 6 hours with a time to achieve the maximum plasma level (54.6 ng/mL) and the GI level (60.1 ng/mL) at half-life times of 30 min in the GI and 1.6 hours in the plasma, respectively. On the other hand, the NC concentration in the GI was superimposed on a gradually increasing level of the plasma concentration starting from a 6-hour time point.

The NC/HP*β*CD concentration in the bloodstream was predicted to be oscillating every 24 hours to achieve the maximum GI level (81.5 ng/mL) and plasma level (41.5 ng/mL) at half-life times of 4 hours in the GI and 5.49 hours in the plasma (exp. value in plasma: 5.49 ± 0.17 hours). The NC/TA*β*CD concentration in the bloodstream was predicted to be oscillating also every 24 hours to reach the maximum plasma level (21.6 ng/mL) and the GI level (28.9 ng/mL) at half-life times of 2.2 hours in the GI and 7.38 hours in the plasma (exp. value in plasma: 7.38 ± 1.61 hours). On the other hand, the complexes did not show sharp peak plasma concentrations similar to those observed by other researchers [1, 26]. All predicted maximum plasma concentrations were in agreement with experimentally-derived data for analyzed substances: 69.64 ± 8.57 ng/mL for NC, 39.65 ± 1.11 ng/mL for NC/HP*β*CD, and 18.25 ± 0.74 ng/mL for NC/TA*β*CD, respectively. The GI and plasma concentration levels for NC and the GI concentrations for NC/HP*β*CD and NC/TA*β*CD after oral administration showed sharp peaks and abrupt decrease of concentration, especially for NC due to a short elimination half-life (1.6 hours). The NC drug kinetics in plasma were already previously described by a similar model revealing a very short *t*_1/2_ of distribution phase [27].

The plasma concentration levels for both formulations were significantly prolonged to 24 hours in retention time because of the improvement of NC dissolution profile upon complexation with cyclodextrins in simulated intestinal or gastric fluids with higher stability constants [24].

In general, the limit cycle graph reflected the observation that the concentration in the circulatory system {*y*(*t*)} was predicted to be an oscillation superimposed without the total gradual increase of concentration. Considering the fact that many of the drug distribution processes in the human body are usually not saturated at normal therapeutic dose levels, the pharmacokinetic-mathematical model used to describe the GI and plasma concentrations was simplified.

In the drug designing process, it is important to keep the concentration as a uniform function to reach the limit cycle as fast as possible. Therefore, the half-life parameters in the GI or plasma can be adjusted to prolong and optimize the “time release” mechanism. The plasma *t*_1/2_ value in our model represents characteristic properties of the analyzed compound. Because the {*y*(*t*)} value is *D*(*t*)- and *x*(*t*)-dependent as the level of the drug in the circulatory system, it should take longer time period for a drug to be effective.

Subsequently, all the studied substances were metabolized in the liver as a result of first-order series of parallel irreversible reactions represented by a linear system of coupled ODE [28].

The dissolution profile of NC/TA*β*CD was determined as 4.38-fold less calculated from the *k*_GI(NC)_/*k*_GI(NC/TA*β*CD)_ ratio than this profile for the NC drug alone. Therefore, initial concentration parameter (*C*_*A*_) was set to *C*_*A*_/4.38. The *C*_*B*_ parameter was set to 2.5 *C*_*B*_ for the same compound.

The process of NC, NC/HP*β*CD, and NC/TA*β*CD conversion into their metabolites might be referred to as an initial value problem because the initial conditions of the dependent variables must have a known *t* which determines how the dependent variable changes with time as shown in Algorithm 2.

The short-range pharmacokinetic profiles of plasma concentrations for analyzed compounds were precalculated to determine maximum plasma concentration (*C*_max⁡_^pre^) and the time to reach it (*T*_max⁡_^pre^), such as 0.58 ng/mL and 0.07 min for NC, 0.43 ng/mL and 0.4 min for NC/HP*β*CD, and 0.18 ng/mL and 0.44 min for NC/TA*β*CD using experimentally-determined rate constants (Table 1). The rate constants were optimized to produce the *C*_max⁡_ values close to the experimentally-determined ones for the analyzed substances, such as *k*_GI(Plasma)_/7.14 for NC, *k*_GI(Plasma)_/4.88 for NC/HP*β*CD, and *k*_GI(Plasma)_/5.38 for NC/TA*β*CD, in comparison to those from the experimental study [1]. Further, these refined rate constants (*k*_GI*_ and *k*_Plasma*_) were implemented in the model to fit both predicted and experimental parameters (Table 2).

**Figure 3:**
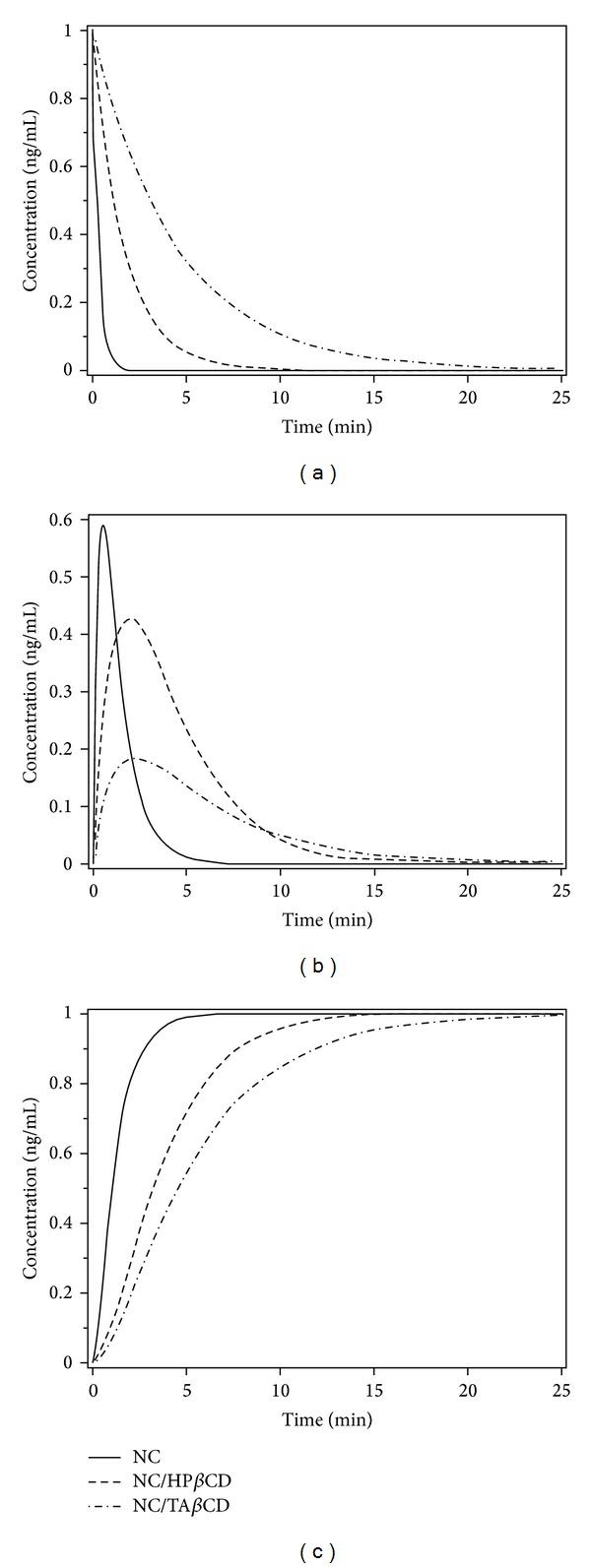
Short-range GI (a) and plasma concentration (b) levels for NC, NC/HP*β*CD, and NC/TA*β*CD and their metabolizing rates (c) as functions of time using refined rate constants.

Since nicardipine undergoes extensive bio-transformation of the N-benzyl side-chain on position 3 of the molecule under liver control and oxidation to the analogous pyridine metabolite [29], it is important to assess and compare the NC metabolizing rates with its cyclodextrin formulated complexes. Under these conditions, the stability of the NC-cyclodextrin complex plays an important role in extending a protection against the oxidation due to the fact that the drug hydrolysis in CD complex is slower [30]. Therefore, to adapt the metabolic body conditions and reach the equilibrium, the absorption of NC in the GI was very rapid resulting in a decrease of its GI concentration level after 2 min, 10 min for NC/HP*β*CD, and 25 min for NC/TA*β*CD formulations (Figure 3(a)). The NC plasma concentration reached its maximum level (0.59 ng/mL) after 0.52 min. After this threshold, the NC plasma concentration was dropped down significantly reflecting drug rapid disappearance in plasma after 5 min. On the contrary, the NC/HP*β*CD and NC/TA*β*CD plasma concentrations were reached with their maximum levels (0.43 ng/mL and 0.18 ng/mL) only after 1.98 and 2.34 min, respectively (Figure 3(b)). Subsequently, they were also dropped down to undetectable levels after 15 and 20 min, indicating the sustained-release behavior of these complexes with significant *t*_1/2_ parameters. This observation was experimentally confirmed as the plasma concentrations of the NC formulations were maintained at a relatively constant level for a long time (up to 24 hours) after their oral administration [1]. The plateau obtained in the NC metabolite concentration rate was more evident even after 5.5 min time in comparison to 15 min for NC/HP*β*CD and 26 min for NC/TA*β*CD (Figure 3(c)). It is interesting to note that the plateau was reached after significant delay time for the NC formulations, which suggested a time increase in their metabolizing rates corresponding to a greater extent of their plasma levels compared to the drug alone. Similarly, the NC plasma peak concentration did not appear after the administration of its complexes, indicating that the side effects may also be reduced. To validate our data with the experimental results, we calculated plasma *C*_max⁡_ values for the analyzed substances taking into account the appropriate *T*_max⁡_: 60 min for NC, 180 min for NC/HP*β*CD, and 240 min for NC/TA*β*CD, respectively. All predicted maximum plasma concentrations were very close to the experimentally-derived values: 68.07 ng/mL (69.64 ± 8.57 ng/mL) for NC, 39.09 ng/mL (39.65 ± 1.11 ng/mL) for NC/HP*β*CD, and 18.46 ng/mL (18.25 ± 0.74 ng/mL) for NC/TA*β*CD.

## 4. Conclusion

The NC release rate is the half-life-dependent process that can be adjusted to prolong and optimize the “time release” mechanism by controlling the formulation ratio of its hydrophilic (NC/HP*β*CD) or hydrophobic (NC/TA*β*CD) complexes. Because {*y*(*t*)} is *D*(*t*)- and *x*(*t*)-dependent as the level of the drug in the circulatory system, it should take a longer period of time for a drug to be effective. With subsequent equal dose levels for specified chemical compounds and when taken orally, the periodic plasma concentrations were significantly prolonged for the NC formulations, especially for its hydrophobic form, and maintained thereafter.

The plasma *t*_1/2_ value in our model represents characteristic properties of the analyzed compound, which affects not just plasma concentration levels of the drug but its metabolizing rates.

## Figures and Tables

**Algorithm 1:**
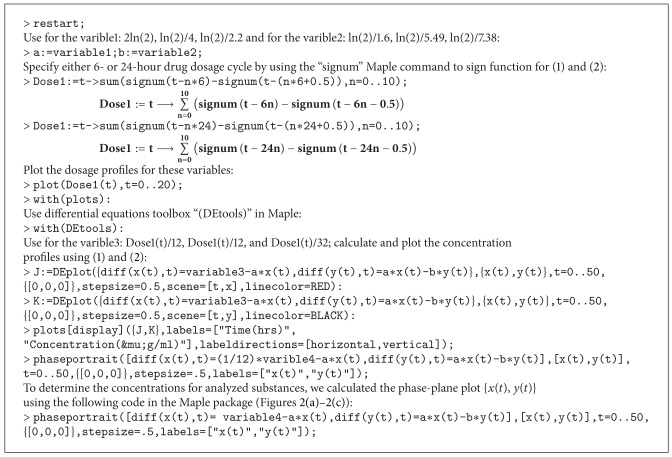


**Algorithm 2:**
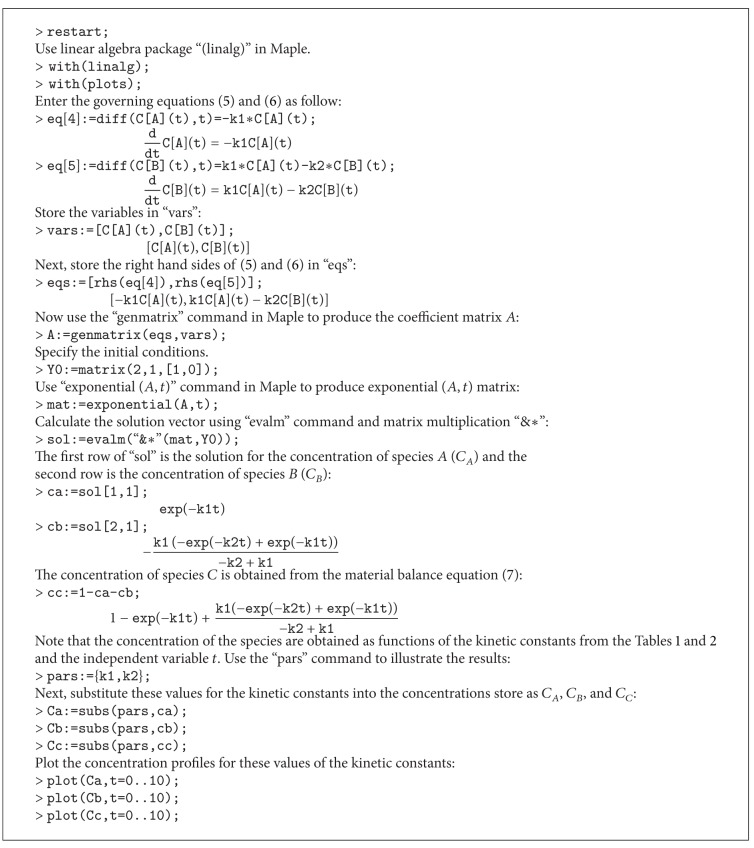


**Table 1:** Rate constants calculated from the predicted (GI) and experimental (Plasma) *t*_1/2_ data [1].

Compound	*k*_GI_ (mL/min)	*k*_Plasma_ (mL/min)
NC	23.1	7.2
NC/HP*β*CD	2.88	2.1
NC/TA*β*CD	5.28	1.56

**Table 2:** Refined rate constants calculated from the predicted (GI) and experimental (Plasma) *t*_1/2_ data [1].

Compound	*k*_GI*_ (mL/min)	*k*_Plasma*_ (mL/min)
NC	3.18	0.99
NC/HP*β*CD	0.59	0.43
NC/TA*β*CD	1.0	0.29

*k*_GI*_ = *k*_GI_/Fold; *k*_Plasma*_ = *k*_Plasma_/Fold; Fold = *T*_max⁡_ *C*_max⁡_^pre^/*T*_max⁡_^pre^ *C*_max⁡_.
